# CART in the regulation of appetite and energy homeostasis

**DOI:** 10.3389/fnins.2014.00313

**Published:** 2014-10-13

**Authors:** Jackie Lau, Herbert Herzog

**Affiliations:** Neuroscience Division, Garvan Institute of Medical ResearchSydney, NSW, Australia

**Keywords:** CART, energy homeostasis, stress response, food intake, body weight

## Abstract

The cocaine- and amphetamine-regulated transcript (*CART*) has been the subject of significant interest for over a decade. Work to decipher the detailed mechanism of CART function has been hampered by the lack of specific pharmacological tools like antagonists and the absence of a specific CART receptor(s). However, extensive research has been devoted to elucidate the role of the CART peptide and it is now evident that CART is a key neurotransmitter and hormone involved in the regulation of diverse biological processes, including food intake, maintenance of body weight, reward and addiction, stress response, psychostimulant effects and endocrine functions (Rogge et al., 2008; Subhedar et al., 2014). In this review, we focus on knowledge gained on CART's role in controlling appetite and energy homeostasis, and also address certain species differences between rodents and humans.

## Introduction

Experiments conducted with acute administration of cocaine or amphetamine in rodents resulted in the upregulation of a particular mRNA species in the striatum of the brain that was subsequently named “cocaine- and amphetamine-regulated transcript” (CART) and the encoded peptides are referred to as CART peptides (Spiess et al., 1981; Douglass et al., 1995). Importantly, CART mRNA levels were also found increased in the nucleus accumbens on post-mortem tissues from human victims of cocaine overdose (Albertson et al., 2004). CART is transcribed as two alternatively spliced mRNAs that are of different lengths and hence produce pro-peptides of different lengths, called proCART 1–89 and proCART 1–102 (Douglass et al., 1995; Douglass and Daoud, 1996). However, the mRNA splicing has no effect on the final peptide, as the active parts of the CART peptides are encoded by a sequence that lies downstream of the spliced region and is therefore identical in both pro-peptides (Douglass et al., 1995; Dey et al., 2003). However, the proCART peptides contain several cleavage sites that allow post-translational processing by pro-hormone convertases in a tissue-specific manner (Seidah et al., 1991; Douglass et al., 1995; Douglass and Daoud, 1996; Dong et al., 1997; Koylu et al., 1997; Steiner, 1998; Kuhar and Yoho, 1999; Thim et al., 1999; Vrang et al., 1999a; Dun et al., 2000; Dey et al., 2003; Stein et al., 2006). This processing produces at least two known biologically active peptides, CART I (55–102) and CART II (62–102), each containing three potential disulphide bridges (Douglass et al., 1995; Thim et al., 1998; Dey et al., 2003; Dominguez, 2006).

CART peptides are evolutionarily strongly conserved between rodent and human (Douglass et al., 1995; Douglass and Daoud, 1996; Adams et al., 1999), with about 95% amino-acid identity between the active neuropeptides (Douglass and Daoud, 1996; Thim et al., 1998; Yermolaieva et al., 2001; Dominguez, 2006), suggestive of a conserved critical physiological function. Notably, high levels of CART expression have been identified to localize in brain regions that include the arcuate nucleus (Arc), the lateral hypothalamus area (LHA), the paraventricular nucleus (PVN), and the nucleus accumbens (Acb) (Douglass et al., 1995; Gautvik et al., 1996; Couceyro et al., 1997; Koylu et al., 1997, 1998; Kristensen et al., 1998; Vrang et al., 1999a; Hubert and Kuhar, 2008; Cavalcante et al., 2011), suggesting an important role for CART in the regulation of food intake and energy homeostasis. This is consistent with results from injections of CART peptide into the nucleus accumbens which have shown an inhibition of feeding in rodents (Yang and Shieh, 2005; Jean et al., 2007). In addition, CART in the Arc is co-localized with α-melanocyte stimulating hormone (α-MSH) (Chronwall, 1985; Adam et al., 2002), which is produced from the proopiomelanocortin (POMC) precursor and is a major inhibitor of appetite and food intake (Fan et al., 1997; Kim et al., 2000; Zheng et al., 2005). In the Arc, CART mRNA levels are regulated by circulating leptin (Kristensen et al., 1998; Schwartz et al., 2000) and are increased by peripheral leptin administration (Elias et al., 1998; Kristensen et al., 1998; Wang et al., 1999; Elias et al., 2001), again indicative of a critical role in energy balance regulation. Moreover, injection of CART I (55–102) into the PVN or Arc of rats markedly enhanced the mRNA expression for the uncoupling protein-1 (*UCP-1*) in brown adipose tissue, relating CART peptides not only to the control of feeding but also the modulation of energy expenditure (Wang et al., 2000; Kong et al., 2003). Mechanistically, it has been shown that application of CART I (55–102) to hypothalamic explants can stimulate the release of corticotropin-releasing hormone (CRH) and thyrotropin releasing hormone, which further links CART to the regulation of the hypothalamic-pituitary-adrenal axis (Stanley et al., 2001). Importantly, several genetic studies have associated mutations or polymorphisms in the *CART* gene in humans with obesity, clearly demonstrating a crucial role of CART in the control of energy homeostasis also in humans (Hager et al., 1998; Challis et al., 2000; del Giudice et al., 2001; Yamada et al., 2002; Dominguez et al., 2004a; Guerardel et al., 2005; Yanik et al., 2006; Rigoli et al., 2010).

While a specific CART receptor(s) has not been identified to date, there is strong evidence that CART signaling can be blocked by pertussis toxin (PTX), indicative of the involvement of an inhibitory G-protein-coupling receptor that couples to G_i/o_ proteins (Yermolaieva et al., 2001; Lakatos et al., 2005; Sen et al., 2007). For example, CART I (55–102) has been described to inhibit voltage-gated L-type Ca^2+^ channels in hippocampal neurons, an effect that was blocked by treatment with PTX (Yermolaieva et al., 2001). Furthermore, central administration of CART I (55–102) stimulated the phosphorylation of cyclic AMP-response-element-binding protein (CREB) in CRH neurons in the PVN of fasted and fed rats (Sarkar et al., 2004), which again is classified as a PTX-sensitive mechanism. Finally, CART I (55–102) application activated extracellular signal-regulated kinase (ERK) phosphorylation in the rodent pituitary-derived cell lines, AtT20 and GH3, and such CART-induced effects were attenuated by a MEK kinase inhibitor as well as pre-treatment with PTX (Lakatos et al., 2005), further supporting the mediation of CART action by inhibitory G proteins.

## Distribution of CART expression

CART can be found in both the central nervous system (CNS) (Spiess et al., 1981; Douglass et al., 1995; Gautvik et al., 1996; Couceyro et al., 1997; Koylu et al., 1997; Smith et al., 1999; Hubert and Kuhar, 2005, 2008; Dominguez, 2006; Vrang, 2006) and the periphery (Koylu et al., 1997; Broberger et al., 1999; Ekblad et al., 2003; Larsen et al., 2003; Wierup et al., 2004; Ekblad, 2006; Vicentic, 2006; de Lartigue et al., 2007; Kasacka et al., 2012). *CART* mRNA-containing neurons are present in high densities in diverse regions such as the Edinger-Westphal nucleus, ganglion cells in the retina, mitral and tufted cells of the olfactory bulb, sensory barrels in the cerebral cortex, the pituitary, lamina X of the spinal cord, medulla of the adrenal cortex, the vagal nuclei, and a number of hypothalamic nuclei (Douglass et al., 1995; Couceyro et al., 1997). Expression of CART in peripheral blood and pituitary portal has also led to the identification of CART-positive neuroendocrine neurons in the hypothalamus, where CART was demonstrated to constitute a releasing factor delivered to the hypothalamic-pituitary-adrenal (HPA) portal circuit for potential endocrine regulation (Larsen et al., 2003; Vicentic, 2006). Consistent with a role in energy homeostasis, CART expression has also been associated with glucose-sensing sites both centrally and peripherally, in both rodents and humans (Jensen et al., 1999; Wierup et al., 2005, 2006, 2007; Kasacka et al., 2012). In the periphery, CART expression has been identified in the islet endocrine cells, ganglionic cells, as well as the sensory and autonomic nerve fibers of the pancreas (Jensen et al., 1999; Wierup et al., 2006, 2007; Kasacka et al., 2012). A recent paper also reported the expression of CART mRNA and protein in subcutaneous and visceral white adipose tissues from both rat and human (Banke et al., 2013), serving a potential novel role in adipocytes by fine-tuning lipolysis and lipase activation to affect lipid- and glucose-homeostasis (Vasseur et al., 2007; Banke et al., 2013).

In addition to the structural conservancy between CART isoforms across species (Douglass et al., 1995; Douglass and Daoud, 1996; Adams et al., 1999; Dey et al., 2003), functional conservation of CART in the mammalian neuroendocrine system has also been implicated in the resembling *CART* mRNA and peptide distribution pattern in the brain observed between humans, rodents, as well as monkeys (Douglass and Daoud, 1996; Gautvik et al., 1996; Charnay et al., 1999; Cavalcante et al., 2011). As a leptin-regulated neurotransmitter with potent appetite-suppressing activity (Kristensen et al., 1998), CART expression in the CNS is highly localized to distinct brain areas critically involved in the control of energy homeostasis, limbic and sensory functions, as well as throughout the HPA axis (Douglass et al., 1995; Gautvik et al., 1996; Koylu et al., 1997; Charnay et al., 1999; Dominguez, 2006; Hubert and Kuhar, 2008; Cavalcante et al., 2011). Localization analyses of CART expression at the mRNA and peptide levels have demonstrated concordance across studies applying cDNA sequencing (Douglass et al., 1995), *in situ* hybridization (Douglass et al., 1995; Couceyro et al., 1997) and immunohistochemistry (Koylu et al., 1997). Indeed, *CART* mRNA has been shown to constitute the third most abundant transcript identified in rat hypothalamus after subtraction of cerebellar and hippocampal mRNAs (Gautvik et al., 1996).

## Central CART in the regulation of appetite and energy homeostasis

A considerable amount of information has been accumulated for the role of CART in modulating metabolism via actions within the CNS. Together with several other critical neuropeptides, CART is integrated into the circuits that control the overall regulation of energy balance. The major site of action is the hypothalamic arcuate nucleus located at the base of the hypothalamus in an area where the blood-brain barrier is semi-permeable, hence uniquely assessable to circulating humoral and metabolic mediators (Barsh and Schwartz, 2002; Berthoud, 2002; Faouzi et al., 2007). As a consequence, the arcuate nucleus circuit serves as sensor of whole-body energy status represented by adiposity levels and promptly directs downstream responses accordingly through neuronal signaling to maintain a constant level of fuel stores (Schwartz et al., 2000; Barsh and Schwartz, 2002).

Two sets of neurons with reciprocal metabolic effects reside in the Arc, namely the orexigenic neuropeptide Y (NPY)/AgRP neurons which promote food intake and reduce energy expenditure, and the adjacent anorexigenic POMC/CART neurons that inhibit food intake and increase energy expenditure (Chronwall, 1985; Elmquist et al., 1998; Barsh and Schwartz, 2002). In response to circulating hormones, such as leptin and insulin that are secreted from adipose tissues and the pancreas, respectively, with a plasma level proportionate to body adipose stores (Zhang et al., 1994; McGregor et al., 1996; Ostlund et al., 1996), hypothalamic expression of the two sets of neurons is differentially regulated (Schwartz et al., 2000). For instance, leptin and insulin levels are reduced following food deprivation that lowers body fat stores, which inhibits POMC/CART neurons and stimulates NPY/AgRP neurons (Schwartz et al., 2000). In addition, the NPY/AgRP neurons are also subject to activation by the circulating orexigenic peptide ghrelin released from the stomach with peak levels before meal initiation (Nakazato et al., 2001).

NPY/AGRP and POMC/CART neurons are not merely two independent sensors mediating opposing effects and projecting parallel extensions to second-order hypothalamic neuropeptide neurons, activity of the two pairs of neurons is subject to regulation by a multitude of mechanisms involved in the control of energy homeostasis. For instance, neuroanatomical studies have shown that there are intra-arcuate connections between the two neuronal subgroups, which are autoregulated by melanocortin peptides as well as local γ-aminobutyric acid (GABA)-mediated effects (Bagnol et al., 1999; Cowley et al., 2001). POMC/CART neurons express the melanocortin 3-receptor (MC3-R) which is specific for melanocortin peptides derived from the post-translational cleavage of POMC (Bagnol et al., 1999), such as the anorectic α-MSH (Fan et al., 1997; Huszar, 1997; Kim et al., 2000; Zheng et al., 2005). The activation of a subset of Arc POMC/CART neurons following exposure to leptin via leptin receptors has been reported to impose an autoinhibitory effect exerted through MC3-R activation in response to elevated release of POMC-derived peptides (Elias et al., 1998; Ahima et al., 1999; Cowley et al., 2001). However, inconsistency exists with the proposed negative autoreceptor manner of MC3-R in diminishing POMC/CART neuronal activity due to the mild increase in adiposity and body weight observed in MC3-R-deficient mice (Butler et al., 2000; Chen et al., 2000). In addition to the potential feedback mechanism described above, the inhibitory neurotransmitter GABA constitutes a principal modulator in the complex intra-arcuate circuit (Cowley et al., 2001). Subpopulations of Arc neurons harbor colocalized expression of NPY and GABA (Horvath et al., 1997), where GABA released from the orexigenic NPY/GABA terminals induces inhibitory post-synaptic currents (IPSCs) that consequently hyperpolarizing hence decreasing firing rate of the post-synaptic POMC/CART neurons (Cowley et al., 2001). Besides such direct neuronal innervations synapsing into Arc POMC/CART neurons by GABA-secreting NPY neurons (Cowley et al., 2001), a recent investigation has demonstrated the direct action of leptin on hyperpolarizing presynaptic GABAergic neurons in reducing GABA-mediated inhibitory tone to POMC/CART cells (Vong et al., 2011), whilst ghrelin was shown to trigger the opposite events on the same neurons (Cowley et al., 2003). Collectively, the electrophysiological regulation of arcuate POMC/CART neurons constitutes two primary mechanisms, including the direct effects of potent neuropeptide modulators on membrane potential through influencing ion channel activity, as well as the indirect impacts on IPSC frequency marking GABA inhibition from local NPY/GABA neurons (Cowley et al., 2001). The resultant resting action potential is purported to concurrently involve the autoregulatory effects of melanocortin peptides (Cowley et al., 2001), ultimately providing an integrated circuit at the Arc for the regulation of food intake and energy homeostasis.

Present in both classic neuroendocrine neurons and hypothalamic projection neurons, Arc CART displays an almost 100% colocalization with POMC and complete segregation from the more medially located orexigenic NPY in rodents (Elias et al., 1998; Vrang et al., 1999a; Fekete et al., 2000, 2004). The co-existence of CART with POMC and derivatives such as α-MSH were found to persist throughout the rostrocaudal extent of the Arc (Vrang et al., 1999a), wherein CART immunoreactivity (IR) was depicted in a separate study to pervade all α-MSH-IR perikarya and axons (Menyhért et al., 2007), which accords with the anorectic role widely adopted for POMC/CART neurons. In accordance with established neuronal projections from Arc (Schwartz et al., 2000), all α-MSH-IR axons in the PVN have also been shown to contain CART immunoreactivity (Menyhért et al., 2007).

In addition to co-storage with the POMC and associated cleavage products which promote negative energy balance (Fan et al., 1997; Huszar, 1997; Kim et al., 2000; Zheng et al., 2005), CART exhibits >95% colocalization with the orexigenic melanin-concentrating hormone (MCH) (Qu et al., 1996; Rossi et al., 1997; Ludwig et al., 1998) at the LHA and zona incerta (ZI) (Vrang et al., 1999a; Elias et al., 2001; Menyhért et al., 2007). All MCH-positive cells showed immunoreactivity for CART in the rostral ZI and the most medial region of LHA, whilst an increasing number of non-CART MCH cells were detected in the caudal and lateral parts of the LHA apart from the CART-containing MCH neurons (Vrang et al., 1999a). In regard to the extensive hypothalamic colocalization of CART-IR with both anorexigenic and orexigenic neurotransmitters, namely POMC at the Arc and MCH at the LHA and ZI respectively, it has been hypothesized that CART co-stored within POMC-IR neurons, functions to counteract the effects of MCH in feeding stimulation, on the assumption that CART and MCH may be co-released (Shimada et al., 1998; Vrang et al., 1999a). This is supported by the observation of an elevated CART tone in the MCH-IR LHA neurons in *MCH* knockout mice, which exhibited anorexic tendencies marked by hypophagia and a leaner phenotype (Shimada et al., 1998; Vrang et al., 1999a). Interestingly, no colocalization was found between CART and another orexigenic peptide confined to neurons in the LHA, orexin B (de Lecea et al., 1998; Peyron et al., 1998; Sakurai et al., 1998; Vrang et al., 1999a), which increases in transcription activity during fasting to elicit stimulatory effects on feeding (Sakurai et al., 1998).

Colocalization experiments exploring the resemblance of CART distribution in feeding-related neuronal groups of the human hypothalamus revealed that, while overlap is seen in certain areas, some striking differences in colocalization pattern also exist (Charnay et al., 1999; Elias et al., 2001; Menyhért et al., 2007). For example, the close overlap of CART and POMC expression in rodents is not so obvious in the human brain (Mihaly et al., 2000; Menyhért et al., 2007). In particular, CART expression could not be detected in the perikarya and axons of α-MSH-IR neurons but interestingly was found in approximately 30% of NPY/agouti-related protein (AgRP) neurons (Mihaly et al., 2000) in the human infundibular nucleus analogous to the Arc in rodents (Menyhért et al., 2007). However, similar co-expression patterns of CART and other neurotransmitters between human and rodents were seen in the PVN region (Menyhért et al., 2007). Intriguingly, the co-expression of CART in NPY/AgRP instead of POMC neurons of the Arc was also observed in other primates such as monkeys (Grayson et al., 2006). Comparable with the observation in rodents, CART was demonstrated to colocalize with MCH in the lateral hypothalamus of humans, particularly in the perifornical region where up to 50% overlap in immunoreactivities of the two peptides was detected (Menyhért et al., 2007). In the infundibular nucleus, fibers comprising MCH-IR were also observed in a portion of CART-IR axons, suggestive of lateral hypothalamic origin (Menyhért et al., 2007).

It is of particular interest that CART in humans has been shown to exhibit the contrasting colocalization with the orexigenic NPY/AgRP neurons and complete segregation from POMC-containing cells in the infundibular nucleus analogous to the rodent arcuate nucleus, whilst co-storage with MCH remained in the lateral hypothalamus. Speculations entailing an orexigenic role of CART as well as the involvement in other functions have therefore arisen, which is supported by the elevated food intake following CART administration to the PVN even in rodents (Stanley et al., 2001). Such paradoxical potentials of CART in feeding behaviors as evident in multiple pharmacological and physiological studies will be discussed subsequently in the present review. With regard to the resembling colocalization with the orexigenic MCH in the LHA consistent across species investigated (Ludwig et al., 1998; Vrang et al., 1999a; Elias et al., 2001; Menyhért et al., 2007), although CART expression appeared unaffected by alterations in energy homeostasis in the MCH neurons in rodents, MCH administration effectively blocked intracerebroventricular CART-induced stimulation of central dopaminergic neurons (Yang and Shieh, 2005). Indeed, lateral hypothalamic CART synthesis has been linked to the regulation of dopaminergic reward pathways (Philpot and Smith, 2006), where psychostimulant-like effects were modulated by CART through moderating the activities of dopaminergic neurons (Jaworski et al., 2003; Dominguez et al., 2004b; Kuhar et al., 2005). Thus, while possibility exists that CART and MCH may participate in regulating different physiological functions effectuated by the same neuronal groups, anatomical and functional interplay of the two peptides has been indicated to prevail in the interactions between food intake and reward neurocircuitries (Saper et al., 2002; Yang and Shieh, 2005; Pang and Han, 2012). Knowledge regarding the underlying cellular mechanisms awaits further extension through neuronal mapping and biochemical studies.

While the role of CART in controlling appetite and energy homeostasis in the human system might be somewhat different, in rodents, the neuronal network in which CART is involved to modulate energy homeostasis has been well-described. The expression of endogenous CART at brain regions involved in feeding regulation has been shown to be sensitive to the energy balance status and the genetic background of mice. In brief, fasting has been documented in various mammals to reduce *CART* mRNA levels at the hypothalamic PVN, Arc, perifornical region, as well as the nucleus accumbens shell (AcbSh) of the striatum, whilst refeeding restored the expression (Kristensen et al., 1998; Lambert et al., 1998; Adams et al., 1999; Vrang et al., 1999a, 2000; Henry et al., 2001; Li et al., 2002; Yang and Shieh, 2005; Van Vugt et al., 2006; Germano et al., 2007). As a leptin-regulated neurotransmitter, the expression of CART mRNA and peptide levels at the Arc is described to be positively correlated with circulating leptin levels (Kristensen et al., 1998; Ahima et al., 1999; Ahima and Hileman, 2000; Rohner-Jeanrenaud et al., 2002; Wortley et al., 2004). This relationship between leptin and CART levels was less consistently demonstrated at the PVN and LHA (Wortley et al., 2004), despite evidence supporting a pivotal role of CART-containing neurons projecting from the Arc to the second order neurons located in the PVN and LHA in producing anorexia (Elias et al., 1999; Wang et al., 2000; Fekete et al., 2004). Intravenous leptin administration was shown to induce Fos expression in hypothalamic CART neurons in the PVN, DMH, Arc, and the ventral premammillary nucleus (Elias et al., 1998, 2001). Whilst Arc CART mRNA and peptide levels were strongly downregulated in food-deprived animals, the transcripts were nearly absent in genetically obese *fa/fa* rats and *ob/ob* mice with disrupted leptin signaling, wherein daily intraperitoneal leptin treatment led to *CART* restoration in the Arc and DMH (Friedman and Halaas, 1998; Kristensen et al., 1998; Ahima et al., 1999; Ahima and Hileman, 2000; Rohner-Jeanrenaud et al., 2002), suggesting that leptin-induced anorexigenic actions may be mediated via CART neurons at the Arc. In comparison, in the *anx/anx* anorexia mouse model characterized by marked reduction in feeding and premature death, *CART* expression was significantly lower in the Arc, and less prominently in the DMH and LHA regions (Johansen et al., 2000). The reduced Arc *CART* expression, together with downregulated serum leptin levels, was attributed to a compensatory response to the energy-deprived state, as well as a probable molecular defect in the Arc deregulating the cellular source of *CART* mRNA (Johansen et al., 2000). In contrast with genetically obese animal models, diet-induced obese (DIO) rodents subjected to a high fat diet (HFD) have been shown to display higher Arc *CART* mRNA levels compared with lean animals fed a low fat diet, due to hyperleptinaemia (Rohner-Jeanrenaud et al., 2002; Wortley et al., 2004; Hou et al., 2010). Elevated *CART* transcript levels were also found in normal-weight obesity-prone rats compared to obesity-resistant subjects, where the Arc leptin-CART pathway was proposed to respond to fat-rich dietary intervention through inhibiting excessive body fat accrual by substituting lipid storage with lipid mobilization (Rohner-Jeanrenaud et al., 2002; Wortley et al., 2004).

Despite purported relevance of CART in the modulation of gastrointestinal (GI) function, it is at present unclear the definite roles of enteric CART in intestinal motility, satiety and feeding behavior (Okumura et al., 2000; Ekblad et al., 2003; Smedh and Moran, 2003; Tebbe et al., 2004). Convincing evidence supporting the GI effects of CART have been provided by independent investigations, where central injections of CART in rodents elicited an anorexigenic response accompanied by the inhibition of gastric emptying and gastric acid secretion (Okumura et al., 2000; Asakawa et al., 2001; Smedh and Moran, 2003), while colonic motility measured by colonic transit time was accelerated (Tebbe et al., 2004). Contrary to a local ENS effect, such alterations in GI functions were indicated to be conveyed through CART acting in the CNS (Okumura et al., 2000; Ekblad et al., 2003; Smedh and Moran, 2003, 2006; Tebbe et al., 2004), as intraperitoneal (i.p.) CART administration failed to reduce food consumption or reproduce similar gastric responses (Okumura et al., 2000; Tebbe et al., 2004; Skibicka et al., 2009). Furthermore, pretreatment with central injection of CRH receptor antagonist prior to central CART administration completely abolished the CART-induced gastric effects, suggesting a central CART-directed CNS modulation of the digestive tract behavioral motor functions via CRH-dependent mechanisms (Okumura et al., 2000; Smedh and Moran, 2003, 2006; Tebbe et al., 2004).

Consistent with a potential CART pathway connecting gut-brain signals in the control of food intake, co-expression of CART and the satiety factor cholescystokinin (CCK)-1 receptors has been detected in the *f* afferent neurons in rodents (Crawley et al., 1991; Moran et al., 1992; Mercer and Beart, 1997; Koylu et al., 1998; Broberger et al., 1999; Dun et al., 2001; Lodge and Lawrence, 2001; Zheng et al., 2002; de Lartigue et al., 2007). Substantially, the synergistic effect of CART and the anorexigenic gut hormone CCK (Crawley and Corwin, 1994) on food intake regulation analogous to that documented for leptin and CCK (Barrachina et al., 1997; Broberger et al., 1999; Morton et al., 2005; Merino et al., 2008) has been demonstrated in animal studies, during which the responses to simultaneous as well as separate central administration of CART peptide and the CCK octapeptide (CCK-8) through cannula implantation (Maletinska et al., 2007) were compared (Volkoff, 2006; Maletinska et al., 2008). In fasted lean mice, the anorexigenic effect induced by CART delivery was significantly enhanced by parallel CCK-8 injection when compared with the administration of each particular peptide alone, while the additive reaction was also shown in an open field test where locomotor activity of the subjects was inhibited (Maletinska et al., 2008). On the contrary, application of the CCK-1 receptor antagonist devazepide blocked CART-induced alteration in food intake (Maletinska et al., 2008). Such long-lasting cooperative action on feeding was speculated to associate in turn with the synergistic effect of leptin on CCK-induced satiety, where CART release from the nodose ganglia was mediated by the interaction of low-affinity vagal CCK-1 receptors and leptin receptors to produce short-term satiety (Maletinska et al., 2008; Heldsinger et al., 2012). Furthermore, 17 and 41% of vagal afferent neurons projecting from the nodose ganglia to the stomach and duodenum respectively were identified to be CART-immunoreactive (Zheng et al., 2002). The vagus has been proposed a mediator of the dorsal hindbrain action of CART on gastric motor function, as indicated by the inhibitory gastric effects of hindbrain intracerebroventricular (i.c.v.) CART microinfusion, which was blocked by subdiaphragmatic vagotomy (Smedh and Moran, 2003, 2006). In particular, the dorsal vagal complex (DVC) was demonstrated to be a target site for both CART and CRH in the suppression of gastric emptying, where effective inhibition occurred in response to the intraparenchymal injection of either peptide into the DVC, at a lower dose compared to that required to elicit a noticeable inhibitory effect by hindbrain i.c.v. administration (Smedh and Moran, 2006).

Reciprocal of a central CART-directed modulation of gastric behavior in feeding regulation, the anorectic actions of central CART have also been proposed to result from stimulation by gut hormones (Hunter et al., 2004). Following food intake, CCK released in the GI tract has been delineated to direct CART release from central vagal afferents, where an abundance of *CCK-1* receptor mRNA expression was detected in the nodose ganglion (Broberger et al., 1999; Hunter et al., 2004). CCK-borne information is then mediated by CART to the hindbrain sites where suppression of food intake is elicited (Broberger et al., 1999; Hunter et al., 2004). However, the role of CART released from vagal afferent terminals in the commissural part of the nucleus of the solitary tract (NTS) was suggested to be minor in mediating vagal satiety signals, as a diminished suppression of food intake was exerted by direct NTS subnuclei CART injections compared to hindbrain i.c.v. CART (Zheng et al., 2001, 2002; Skibicka et al., 2009). Thus, the potential function of CART on satiation may involve a distal site of action as well as interaction with other nutritional transmitters critical for vagally-mediated gastrointestinal satiety (Broberger et al., 1999; Zheng et al., 2002). For instance, induction of CART immunoreactivity was readily demonstrated upon CCK administration both in rats and cultured vagal afferent neurons in a state of energy restriction, while refeeding of starved animals markedly increased CART immunoreactivity via a CCK-1 receptor antagonist-sensitive mechanism (de Lartigue et al., 2007, 2010). In addition, introduction of the orexigenic peptide ghrelin inhibited CCK-mediated CART stimulation (de Lartigue et al., 2007), illustrating an interplay between gastrointestinal orexigenic and anorexic peptides in modulating CART expression. In particular, immunoreactive CART neurons have been shown to display differential quantities during developmental changes in the dorsal motor nucleus of the vagus (DMNV), wherein vagal preganglionic neurons are responsible for regulating various ingestive behaviors through innervations in the GI tract (Dun et al., 2001; Zheng et al., 2002). Such descending expression levels upon maturation indicate a potential signaling role of CART critical in the early post-natal development (Dun et al., 2001).

## Peripheral CART in the regulation of energy homeostasis

The proposed physiological role of CART as an endogenous anorexigenic factor was originally deduced from the inhibition of food consumption observed in animal models following hypothalamic or intracerebroventricular administration of CART-derived peptides (Kristensen et al., 1998; Vrang et al., 1999b; Larsen et al., 2000; Volkoff and Peter, 2000; Aja et al., 2001a,b; Stanley et al., 2001; Nakhate et al., 2011, 2013). Based on the dual involvement in both hypothalamic modulation of feeding behavior and autonomic control of gastrointestinal functions common to many neuropeptides, considerable efforts have subsequently been devoted to the investigation of CART-mediated effects in the enteric nervous system (ENS) (Couceyro et al., 1998; Kuhar and Yoho, 1999; Murphy et al., 2000; Okumura et al., 2000; Ekblad et al., 2003; Ellis and Mawe, 2003; Smedh and Moran, 2003; Tebbe et al., 2004). Extensive CART expression has been characterized in the ENS of diverse mammals by *in situ* hybridization (Ekblad et al., 2003) and immunoassays (Couceyro et al., 1998; Kuhar and Yoho, 1999; Murphy et al., 2000; Ellis and Mawe, 2003; Kasacka et al., 2012), affirming the presence of CART mRNA and peptides in nerve cell bodies and fibers innervating the stomach, small and large intestines of the gastrointestinal tract (Ekblad, 2006), particularly within the neuroendocrine cells and myenteric plexus (Kasacka et al., 2012). Such brain-gut CART expression suggests that CNS control of feeding and satiety may be coordinated with local gastric CART-induced effects to produce an integrated regulation of body weight. This is supported by the central CART immunoreactivity profile, which depicts concentrated expression at the hypothalamic nuclei (Spiess et al., 1981; Douglass et al., 1995; Gautvik et al., 1996; Couceyro et al., 1997; Koylu et al., 1997; Smith et al., 1999; Hubert and Kuhar, 2005, 2008; Dominguez, 2006; Vrang, 2006) constituting major relays linking the sensory, motor and limbic areas between forebrain and hindbrain through widespread reciprocal networks, orchestrating autonomic, endocrine and behavioral activities (Fekete et al., 2000; Balkan et al., 2001; Williams et al., 2001; Tebbe et al., 2004). Specifically, anatomical implications of CART indicated by enteric CART expression in digestive function have been further indicated by CART immunoreactivity at the hypothalamic Arc and PVN neurons, as well as brainstem nuclei such as NTS and parabrachial nucleus (PBN), both involved in the efferent and afferent control of GI function through neuropeptidergic mechanisms engaging the complex neuroendocrine and autonomic pathways (Kristensen et al., 1998; Fekete et al., 2000; Aja et al., 2001b; Stanley et al., 2001; Zheng et al., 2001; Tebbe et al., 2003, 2004). In addition, CART expression in the cholinergic neurons of the myenteric plexus and the pancreatic islets further denoted the potential gastric effects of CART conducted via peripheral receptor targets composing the peripheral cholinergic pathways (Couceyro et al., 1998; Jensen et al., 1999; Ekblad et al., 2003; Tebbe et al., 2004; Wierup et al., 2004).

The precise functions of CART peptides released by enteric CART-expressing neurons in the ENS are yet to be determined (Ekblad, 2006). There has been a lack of direct evidence regarding a role of locally produced CART in classical neurotransmission within the GI tract, where intestinal motility as measured by contractile or relaxatory responses was unaffected by CART peptide application when motor activity studies were performed *in vitro* on muscle strips from stomach, small and large intestine (Ekblad et al., 2003). Notably, exceptions to the above may include specific CART-evoked colonic responses, such as the attenuation of nitric oxide-induced intestinal relaxation (Ekblad et al., 2003; Ekblad, 2006), as well as the apparent stimulation of colonic transit, an indirect measure of colonic motor function (Tebbe et al., 2004). In spite of the confined documentation of CART involvement in brain-gut interaction and the indeterminate functional role of enteric CART, accumulating evidence propose a role of CART in intestinal adaptation, where the survival and maintenance of enteric neurons is promoted (Ekblad et al., 2003; Ekblad, 2006). Such neuroprotective property (Ekblad, 2006; Zhang et al., 2012) and intestinal plasticity has been inferred from upregulated CART expression and increased CART-expressing neurons in atrophic intestine and cultured myenteric neurons respectively, conditions indicative of neuronal stress or injury (Ekblad et al., 2003; Ekblad, 2006). In sum, gastric involvement of CART has been evident through discrete histological and physiological experiments, whilst further detailed characterization of distribution and functions may contribute to a comprehensive understanding of the specific roles of enteric CART.

## Functional implications of CART on energy metabolism from pharmacological interventions

The identification of the underlying mechanisms by which CART exerts effects on feeding and energy homeostasis have been challenging due to the lack of any knowledge of the corresponding CART receptor (s) and the absence of specific antagonists. Nevertheless, numerous studies incorporating both pharmacological and genetic manipulations of CART expression in murine models have been endeavored in the last decades to determine the sites of action and the effects on feeding behavior and metabolism of the peptide. Overexpression studies to discern the brain regions mediating CART-induced regulation of feeding have been the most common approach in rodents. Widely adopted as an appetite-regulating peptide of the CNS with hypothalamic expression levels modulated by nutritional status (Kristensen et al., 1998; Thim et al., 1998, 1999; Schwartz et al., 2000), CART was administrated i.c.v. to address the effects of overexpression during varying energy states (Kristensen et al., 1998; Lambert et al., 1998; Thim et al., 1998; Vrang et al., 1999b; Edwards et al., 2000; Kask et al., 2000; Larsen et al., 2000; Aja et al., 2001a,b; Bannon et al., 2001; Nakhate et al., 2011, 2013) (Table 1). The lateral ventricle (LV) of the forebrain or the 3rd ventricle (3V) have been the major injection targets. I.c.v. injection of recombinant CART peptide has been consistently demonstrated to inhibit food intake and body weight gain in a dose-dependent manner in both food-restricted and free-feeding conditions as well as both under standard chow or a nutritionally complete liquid diet, in either normal or diet-induced obese animals (Kristensen et al., 1998; Lambert et al., 1998; Thim et al., 1998; Vrang et al., 1999b; Edwards et al., 2000; Kask et al., 2000; Larsen et al., 2000; Abbott et al., 2001; Aja et al., 2001a,b; Bannon et al., 2001; Rohner-Jeanrenaud et al., 2002; Tachibana et al., 2003; Wortley et al., 2004; Qing and Chen, 2007; Nakhate et al., 2011, 2013). Furthermore, the catabolic capacity of CART appeared sufficient to prevent and attenuate the orexigenic effects of NPY, as i.c.v. and intra-PVN CART potently suppressed feeding in satiated rats subjected to NPY-induced hyperphagia (Kristensen et al., 1998; Lambert et al., 1998; Vrang et al., 1999b; Wang et al., 2000; Rohner-Jeanrenaud et al., 2002). Similarly, the anorectic potency of CART has also been demonstrated in a recent study focusing on the interaction between CART and the GABA type A receptor (GABA-A) active neurosteroid allopregnanolone (ALLO) and the inhibitor neurosteroid dehydroepiandrosterone sulfate (DHEAs) (Nakhate et al., 2013). It was shown in rodents that pre-treatment of i.c.v. CART effectively attenuated subcutaneous ALLO-induced hyperphagia and weight gain, as well as potentiating DHEAS-induced hypophagic and weight reducing effects (Nakhate et al., 2013).

**Table 1:** **Summary of the metabolic and behavioral effects of central CART administration via various intracerebroventricular and intranuclear delivery methods**.

**Year**	**Publication**	**Targeting peptide/CART fragment**	**Route of adminis-tration**	**Species (genetic background)**	**Diet**	**Feeding behavior and body weight alterations**	**Locomotor behavior**
1998	Kristensen et al., 1998	CART I (55–102)	i.c.v.	Rat	Standard chow	↓ Spontaneous feeding; ↓ Fast-induced feeding; ↓ NPY-induced feeding	N/A
1998	Lambert et al., 1998	CART (55–59)	i.c.v.	Rat	Standard chow	~ Spontaneous feeding	N/A
1998	Lambert et al., 1998	CART (55–76)	i.c.v.	Rat	Standard chow	↓ Spontaneous feeding; ↓ NPY-induced feeding	N/A
1998	Lambert et al., 1998	CART (62–76)	i.c.v.	Rat	Standard chow	↓ Spontaneous feeding	N/A
1998	Thim et al., 1998	CART I (55–102); CART II (62–102)	i.c.v.	Mouse	Standard chow	↓ Fast-induced feeding	N/A
1999	Vrang et al., 1999b	CART I (42–89)	i.c.v.^g^	Rat	Standard chow	↓ Spontaneous feeding (in food-restricted animals) and ↓ NPY-induced feeding	N/A
2000	Edwards et al., 2000	CART I (55–102)	i.c.v.	Rat	Standard chow	↓ Spontaneous feeding	N/A
2000	Kask et al., 2000	CART (62–76)	i.c.v.	Rat	Standard chow	↓ Spontaneous feeding	N/A
2000	Volkoff and Peter, 2000	CART I (55–102); CART (62–76)	i.c.v.	Goldfish	Standard chow	↓ Spontaneous feeding and ↓ NPY-induced feeding (in food-restricted animals)	N/A
2000	Larsen et al., 2000	CART I (42–89)	i.c.v. (chronic infusion)	Lean and obese Zucker (*fa/fa*) rats	Standard chow	↓ Spontaneous feeding (in both free-feeding and food-restricted animals); dose-dependent ↓ in body weight	Dose-dependent motor disturbances (combined gait and walking ataxia)
2000	Okumura et al., 2000	CART I (55–102)	i.c.	Rat	Standard chow	↓ Fast-induced feeding^b^	N/A
2000	Wang et al., 2000	CART I (55–102)	intra-PVN	Rat	Standard chow	↓ NPY-induced feeding^i^	N/A
2001	Bannon et al., 2001	CART I (55–102); CART II (62–102)	i.c.v.	Mouse	Standard chow	↓ Fast-induced feeding	N/A
2001	Abbott et al., 2001	CART I (55–102)	Hypothalamic intranuclear injections (VMH, Arc, PVN, SON, DMH, LHA and AHA)	Rat	Standard chow	↑ Spontaneous feeding (only measured in satiated animals cannulated into the DMH or Arc); ↑ Fast-induced feeding	N/A
2001	Abbott et al., 2001	CART I (55–102)	i.c.v. (3V)	Rat	Standard chow	↓ Spontaneous feeding; ↓ Fast-induced feeding (↓ feeding episodes)	Behavioral abnormalities marked by reduced feeding episodes, flat-backed posture and movement-associated tremors (behavioral analysis performed for 24-h fasted animals only but not satiated animals)
2001	Zheng et al., 2001	CART I (55–102)	i.c.v. (LV and 4V)	Rat	Sucrose solution or standard chow	↓ Spontaneous feeding (↓ short-term sucrose intake and ↓ overnight chow intake)—effects more pronounced in 4V compared to LV administration	Alterations in motor behavior (mild movement-associated tremors in part of 4V injected subjects)
2001a	Aja et al., 2001a	CART I (55–102)	i.c.v.	Rat	Ensure liquid diet	↓ Spontaneous feeding (↓ liquid diet intake in licks and meal size in food restricted animals)	Altered oral motor function and behavioral alterations (trance-like state, flat-backed and arched-backed postures, cage licking, movement-associated tremors)
2001b	Aja et al., 2001b	CART I (55–102)	i.c.v. (3V)	Rat	Ensure liquid diet	↓ Spontaneous feeding (↓ liquid diet intake and observations of feeding in food restricted animals)—reductions significantly attentuated by aqueduct obstruction^a^	Alterations in motor behavior (flat-backed and arched-backed postures and movement-associated tremors)—alterations significantly attentuated by aqueduct obstruction^a^
2001b	Aja et al., 2001b	CART I (55–102)	i.c.v. (4V)	Rat	Ensure liquid diet	↓ Spontaneous feeding (↓ liquid diet intake and observations of feeding in food restricted animals)—reductions unaffected by aqueduct obstruction^a^	Alterations in motor behavior (flat-backed and arched-backed postures and movement-associated tremors)—alterations unaffected by aqueduct obstruction^a^
2002	Aja et al., 2002	CART I (55–102)	i.c.v. (4V)	Rat	Ensure liquid diet	↓ Spontaneous feeding (↓ liquid diet and water intake in food restricted animals); production of conditioned taste aversion	N/A
2002	Rohner-Jeanrenaud et al., 2002	CART I (55–102)	i.c.v. (chronic infusion)	Rat (normal and DIO)	Standard chow or HFD	↓ Spontaneous feeding and ↓ NPY-induced feeding; ↓ body weight gain	N/A
2002	Zheng et al., 2002	CART I (55–102)	i.c.v. (4V)^h^ and intra-NTS	Rat	Sucrose solution or standard chow	↓ Spontaneous feeding (↓ short-term sucrose intake)—effects more pronounced in 4V compared to intra-NTS administration	N/A
2003	Smedh and Moran, 2003	CART I (55–102)	i.c.v. (4V)	Rat	Sucrose solution	↓ Spontaneous feeding (↓ sucrose intake in food restricted animals); altered lick microstrcuture parameters^c^	N/A
2003	Kong et al., 2003	CART I (55–102)	intra-Arc^d^	Rat	Standard chow	↑ Spontaneous feeding (in both free-feeding and food-restricted animals) and ↑ Fast-induced feeding; ↑ cumulative body weight gain; ↑ body weight loss following 24-hr fasting and food restriction^i^	N/A
2004	Wortley et al., 2004	CART I (55–102)	i.c.v. (3V)	Rat	Standard chow	↓ Spontaneous feeding	N/A
2005	Yang et al., 2005	CART I (55–102)	intra-AcbSh	Rat	Standard chow	↓ Spontaneous feeding; ↓ Fast-induced feeding; ↓ GABA-A agonist muscimol-induced feeding	N/A
2007	Qing and Chen, 2007	rat CART cDNA	i.c.v.^e^	Rat (DIO)	High fat/high sucrose diet	↓ Spontaneous feeding; ↓ Fast-induced feeding; ↓ body weight gain (↓ lean mass; fat mass unaffected)	N/A
2007	Jean et al., 2007	CART I (55–102)	intra-AcbSh	Mouse	Standard chow	↓ Fast-induced feeding	N/A
2007	Jean et al., 2007	CART siRNA	intra-AcbSh	Mouse	Standard chow	↑ Spontaneous feeding and ↓ stimulating 5-HT_4_R- or MDMA-induced anorexia in staved animals	N/A
2008	Smith et al., 2008	rAAV encoding full length rat CART cDNA (GenBank accession no. U10071)	intra-PVN^e^	Rat	Standard chow or HFD	↑ Cumulative feeding and cumulative body weight gain; effects more accentuated on HFD	N/A
2009	Skibicka et al., 2009	CART I (55–102)	i.c.v. (4V) or intra-NTS	Rat	Standard chow	[4V injection]^f^ ↓ Spontaneous feeding and body weight (in food-restricted animals); hypophagic response and weight loss attenuated by pre-treatment with hindbrain delivery of GLP-1R antagonist (exendin-9-39); intra-NTS injection produced no observable effect on feeding or body weight	N/A
2010	Hou et al., 2010	CART I (55–102)	intra-Arc; intra-DMH	Streptozotocin-diabetic rats	Standard chow or HFD	Chow diet: ↑ Spontaneous feeding (in satiated animals) (Arc) and ↑ Fast-induced feeding (DMH and Arc); HFD: ↑ Spontaneous feeding (Arc)	N/A
2011	Nakhate et al., 2011	CART I (54–102)	i.c.v.	Rat	Standard chow	↓ Spontaneous feeding and body weight; attenuated social isolation-induced hyperphagia and body weight gain	N/A
2013	Nakhate et al., 2013	CART I (54–102)	i.c.v.	Rat	Standard chow	↓ Spontaneous feeding; attenuated ALLO-induced hyperphagia and weight gain; potentiated DHEAS-induced anorexia and weight loss	N/A

*CSF, cerebrospinal fluid; DIO, diet-induced obese; HFD, high fat diet; BAT, brown adipose tissue; i.c.v., intracerebroventricular; i.c., intracisternal; LV, lateral ventricle; 3V, third ventricle; 4V, fourth ventricle; Acb, nucleus accumbens; AcbSh, nucleus accumbens shell; AHA, anterior hypothalamic area; Arc, arcuate nucleus; DMH, dorsomedial nucleus; LHA, lateral hypothalamic area; NTS, nucleus of the solitary tract; PBN, parabrachial nucleus; PVN, paraventricular nucleus; SON, supraoptic nucleus; VMH, ventromedial hypothalamic nucleus*.

*Effects on body weight described where results presented, otherwise either unaffected or information unavailable*.

*Effects on locomotor behavior described where results presented, otherwise either unaffected or information unavailable*.

a *Cerebral aqueduct occlusion to interrupt forebrain-hindbrain CSF flow*.

b *Inhibition of gastric function (suppression of gastric acid secretion and gastric emptying); inhibition of gastric acid secretion remained in vagotomized animals; inhibition of gastric acid secretion blocked by pretreatment with central administration of CRF receptor antagonist α-helical CRF9-41*.

c *Inhibition of gastric function (suppression of gastric emptying); inhibition of gastric emptying blocked by pretreatment with central administration of CRF receptor antagonist α-helical CRF9-41; CART-induced inhibition of gastric emptying proposed unlikely to contribute to CART-mediated inhibition of food intake*.

d *Acute administration through repeated injections and chronic overexpression using stereotactically targeted gene transfer*.

e *Chronic overexpression using recombinant adeno-associated virus vector containing rat CART cDNA*.

f *↑ Blood glucose levels; hyperglycemic response not altered by GLP-1R blockade in animals pre-treated with GLP-1R antagonist (exendin-9-39)*.

g *Induction of Fos expression in the PVN, DMH, SON and Arc (hypothalamus), central nucleus of amygdala (cerebrum), PBN and NTS (hindbrain)*.

h *Induction of Fos expression in NTS neurons*.

i *↑ UCP-1 expression thermogenic capacity in BAT*.

CART administration via the i.c.v. route was also able to eliminate the increase in feeding and deleterious weight gain caused by social isolation in rats (Nakhate et al., 2011), a consequence of the downregulation of the hypothalamic CART-containing system in various hypothalamic feeding-related areas caused by this condition (Nakhate et al., 2011). In the same study, whilst re-socialization of the isolation-reared rats restored the food intake, body weight, and hypothalamic CART-immunoreactivity back to controls levels, immunoneutralization of endogenous CART by i.c.v. CART antibody attenuated the restoration, confirming the important role of CART in feeding regulation under chronic psychological stress condition (Nakhate et al., 2011). This is consistent with other studies that used antibodies raised against different CART segments for blocking central CART signaling, where all of which were able to neutralize the anorectic property of CART and lead to a significant hyperphagic response (Kristensen et al., 1998; Lambert et al., 1998; Nakhate et al., 2010, 2011). In addition to eliciting an anorectic response, gastrointestinal effects including inhibition of gastric acid secretion and gastric emptying have also been reported as a result from i.c.v. CART (Okumura et al., 2000; Asakawa et al., 2001; Smedh and Moran, 2003, 2006; Tebbe et al., 2004). Chronic overproduction of *CART* mRNA via viral approaches or continuous infusion of recombinant CART peptide transferred through i.c.v. cannulas into genetically (*fa*/*fa*) (Larsen et al., 2000) or diet-induced (Rohner-Jeanrenaud et al., 2002; Qing and Chen, 2007) obese rats induces hypophagic effects during fed states and reduced hyperphagia following fasting were also observed. Such reduction in energy intake was accompanied by suppression of body weight gain mainly due to decrease in lean mass (Larsen et al., 2000; Qing and Chen, 2007), indicating the potential of CART in the long-term regulation of food consumption and body mass, under both normal condition and nutritionally induced obesity.

In conjunction with the characterization of physiological responses, neuronal activities stimulated by central CART has been investigated by structural studies for the purpose of identifying brain areas potentially crucial for CART-induced anorectic effects. Following i.c.v. CART administration, temporal expression patterns of the immediate early gene *c-Fos* (Dragunow and Faull, 1989; VanElzakker et al., 2008), which has been adopted to depict neuronal firing of actions potentials (Dragunow and Faull, 1989; VanElzakker et al., 2008), were found to concentrate in the hypothalamic and brainstem structures implicated in the central regulation of feeding (Vrang et al., 1999b; Zheng et al., 2002). In the hypothalamus in particular, high density of Fos expression was located in the PVN and the posterior DMH, while considerable Fos-IR cells were also identified in the Arc and SO. In the brainstem, Fos-positive cell nuclei were also concentrated in the PBN and, more importantly, in the NTS, which serves a key sensory relay nucleus with reciprocal connections with numerous forebrain and brainstem structures (Vrang et al., 1999b). Such CART-induced Fos activation in the NTS has been indicated independent from possible secondary effects triggered by chemo-activation at the area postrema (AP) directed to the NTS, as the chemosensitive neurons in the AP were devoid of Fos-IR cells (Vrang et al., 1999b). Moderately high Fos expression was also detected in cerebral nuclei associated with autonomic functions and energy balance (Smith and DeVito, 1984; Vrang et al., 1999b), including the central nucleus of the amygdala, where neuronal projections also reciprocally link with the PVN of the hypothalamus and the PBN and NTS of the hindbrain (Hopkins and Holstege, 1978; Holstege et al., 1985). The widespread Fos expression pattern elicited by forebrain i.c.v. CART has been demonstrated to encompass an anatomical continuum of neuronal activations across the cerebrum, hypothalamus and brainstem (Vrang et al., 1999b). The paralleled effects on appetite inhibition and metabolic regulation are believed to portray an integrated outcome of the interactions between central CART-interfered pathways residing primarily within the hypothalamic and brainstem neurons. For instance, as aforementioned, the administration of CART combined with other neuromodulatory such as CCK in mice generated synergistic effects on food intake and locomotion, while displaying concomitant enhancement in the number of Fos-positive neurons compared to injecting each peptide alone (Maletinska et al., 2008; Pirnik et al., 2010). The additive effect on Fos immunoreactivity was especially notable in the target areas common to both peptides, namely the hypothalamic PVN, DMH, VMH and Arc, as well as NTS at the brainstem (Maletinska et al., 2008; Pirnik et al., 2010), wherein the CCK-related satiety signals transmitted to the hindbrain were suggested to be further regulated by leptin action integrated in the Arc as well as neuronal signals from both PVN and LHA (Broberger, 2005; Morton et al., 2005; Maletinska et al., 2008).

CART is widely expressed in the brain and particularly concentrated in the hypothalamus, suggestive of a diverse range of functions. While effective, delivery of ligands via the i.c.v. route is associated with the downside of the simultaneous stimulation of pathways in various parts of the brain, likely contrasting with effects attributable to the activation of specific neuronal populations. One such case are the CART neurons at the Arc, which respond to and are modulated by leptin signals, leading to the activation of selective neurons and associated downstream pathways (Kristensen et al., 1998; Schwartz et al., 2000). It is therefore unsurprising that the observed effects of the i.c.v. injection of substances like CART are not always replicated by targeted delivery of the same peptide into specific nuclei of the hypothalamus. Indeed, several studies have shown that targeting CART into individual hypothalamic nuclei results in revelation of the orexigenic effects of CART, leading to increased food intake and body weight (Abbott et al., 2001; Kong et al., 2003; Smith et al., 2008; Hou et al., 2010). The strongest orexigenic effects were observed by injection of CART into the VMH, DMH and Arc, and a much lesser effect was observed when administered into the PVN, LHA, anterior hypothalamic area, and SO (Abbott et al., 2001; Kong et al., 2003; Smith et al., 2008; Hou et al., 2010) (Table 1). Other effects following Arc and PVN CART delivery such as greater energy expenditure and thermogenic capacity, as indirectly measured by the expression and activity of UCP-1 in brown adipose tissue crucial in thermogenesis, has also been reported (Wang et al., 2000; Kong et al., 2003) (Table 1).

Despite the dependence of endogenous CART expression on nutritional states discussed above, energy states of the animals or dietary options appeared to have little influence over the potency of CART administration-induced feeding stimulation (Abbott et al., 2001; Kong et al., 2003; Smith et al., 2008; Hou et al., 2010). For instance, intra-arcuate delivery of CART resulted in elevated food intake in rodents under both fasted, food-restricted and satiated conditions, subjected to the dietary interventions of either regular chow or HFD (Abbott et al., 2001; Kong et al., 2003; Hou et al., 2010). Intriguingly, the orexigenic effects of CART were exhibited in both non-diabetic normal rats as well as streptozotocin-induced diabetic rats, where the intra-Arc CART-induced increase in feeding was reproduced under various energy states and dietary treatments (Hou et al., 2010). Similarly, in rats receiving chronic overexpression of recombinant CART virally delivered into the PVN, higher cumulative food intake and body weight gain was observed in both groups fed either normal chow or HFD compared to control groups, with more pronounced changes in the HFD group (Smith et al., 2008). Underlying such observations, the appetite-promoting effects of hypothalamic intranuclear CART administration may be attributed to a role of hypothalamic CART in stimulating the release of orexigenic neuropeptides locally (Smith et al., 2008; Hou et al., 2010). This is supported by experiments involving a static incubation system where an increase in the release of NPY- and AgRP-IR but not α-MSH-IR was detected in both Arc-containing hypothalamic explants incubated with CART peptide *in vitro* as well as in PVN-containing hypothalamic explants isolated from animals subjected to intra-PVN CART injection (Smith et al., 2008; Hou et al., 2010). Direct hypothalamic intranuclear CART injection at specific sites, therefore resulted in feeding behaviors opposite to the anorectic effects seen for i.c.v. CART.

The discrepancy between the anorexic effects of CART when injected i.c.v. vs. the predominately orexigenic effects of CART when delivered into specific hypothalamic nuclei suggests that CART expression/function in other brain areas may also be important to the regulation of food intake and energy homeostasis, also suggesting that CART may be involved in both anorexigenic and orexigenic circuits in the CNS (Parker and Bloom, 2012). Other potential areas for CART-mediated anorexic effects include the striatum, which is known to have upregulated *CART* expression following acute i.p. administration of psychostimulants (Douglass et al., 1995; Fagergren and Hurd, 1999; Hubert and Kuhar, 2008), reduced *CART* mRNA levels following fasting (Kristensen et al., 1998; Adams et al., 1999; Yang and Shieh, 2005), and has been shown to be involved in the mediation of reward and reinforcement (Koob and Bloom, 1988; Salamone, 1996; Upadhya et al., 2012) as well as the neuronal circuits controlling feeding behavior (Bakshi and Kelley, 1993; Gilbert and Cooper, 1995; Pothos et al., 1995; Stratford et al., 1997; Stratford and Kelley, 1999; Baldo et al., 2002; Yang and Shieh, 2005; Upadhya et al., 2012). Evidence for such a role was gained from experiments in a strain of CCK-1 receptor-deficient obese rats, where a significant reduction in CART immunoreactivity in the Arc was found potentially associated with a diminished anorectic effect of CART peptide compared to lean controls (Abraham et al., 2009). Furthermore, intra-accumbal CART peptide injection has been demonstrated to diminish both basal food consumption and food deprivation-induced feeding (Yang and Shieh, 2005; Jean et al., 2007), as well as potently attenuating the orexigenic effects of the GABA-A agonist muscimol (Yang and Shieh, 2005), albeit some inconsistency across different studies (Jaworski et al., 2008) (Table 1). The antagonistic effects of the GABA system and CART at the Acb were also demonstrated in the neurochemical phenotypes of hypothalamic neurons after the appetite-inducing microinjection of muscimol into the AcbSh, which increased Fos expression in orexin neurons at the perifornical area and NPY neurons at the Arc, while inhibiting that in Arc CART/POMC neurons (Zheng et al., 2003). In a recent study, subcutaneous injection of the GABA-A active neurosteroid ALLO significantly reduced CART immunoreactive cells and fibers in the AcbSh, as well as in other feeding-related hypothalamic nuclei such as the PVN, Arc and LHA (Nakhate et al., 2013). Direct CART administration into the Acb performed by an independent group generated no detectable influence on food reward assessed by food self-administration, yet triggered inhibitory effects on cocaine self-administration, an alternative measure of reward and reinforcement entailing dopaminergic functions (Jaworski et al., 2008).

The anorexia elicited by intra-accumbal CART was more sustainable in freely fed compared to starved animals, highlighting the significance of fuel status on CART function in feeding modulation (Yang and Shieh, 2005). Furthermore, complementary to the overexpression experiments, RNA-interference has been employed to investigate the effects of CART depletion in rodents (Jean et al., 2007; Job and Kuhar, 2012). Tissue-specific CART knockdown in the Acb via intra-accumbal administration of short interfering RNA (siRNA) or short hairpin (shRNA) against *CART* mRNA induced body weight gain and hyperphagia in fed mice (Jean et al., 2007; Job and Kuhar, 2012), as well as abolishing the anorectic effects of serotonin (5-hydroxytryptamine, 5-HT) 4 receptor (5-HT4R) stimulation as well as 3,4-*N*-methylenedioxymethamphetamine (MDMA, ecstasy) treatment in starved mice, further denoting the potential role of Acb CART in mediating the appetite suppressant properties in models of anorexia nervosa (Jean et al., 2007).

Despite the body of evidence endorsing the plausibility of the Acb as a site for CART-directed anorexia, the appetite-regulating effects produced by intra-accumbal CART likely represent part of the reward and motivational responses derived from an interaction between CART and the dopaminergic system in the Acb. Multiple lines of evidence have suggested an inhibitory role of endogenous accumbal CART in addiction-relevant behaviors, which are speculated to act in concert with feeding modulation as well as the locomotive effects mediated by the dopaminergic circuits (Kim et al., 2003; Jaworski et al., 2008; Hubert et al., 2010; Job and Kuhar, 2012; Upadhya et al., 2012). For instance, substantial innervations have been described for CART-containing neurons in the ventral pallidum, a key nucleus harboring accumbal efferents, where CART-IR terminals were reported to compose symmetric synapses resembling inhibitory GABAergic synapses (Hubert et al., 2010). Whilst intra-accumbal administration of CART alone produced no effect on locomotor activity, co-injection with cocaine or amphetamine into the Acb inhibited the cocaine-like locomotor effects produced by Acb dopamine microinfusions, both intra-accumbal and intra-pallidal injections of CART peptide led to reduction in cocaine- and amphetamine-induced locomotor activity (Jaworski et al., 2003, 2008; Kim et al., 2003; Hubert et al., 2010). Correspondingly, CART depletion through intra-accumbal CART shRNA increased cocaine-mediated locomotion (Job and Kuhar, 2012), supporting an antagonistic property of Acb CART in the functions of cocaine and other psychostimulants.

The hindbrain, as a region described to convey post-prandial satiety effects to the hypothalamus, has been considered a potential candidate site for CART action (Marty et al., 2007; Subhedar et al., 2014). Supporting evidence include the moderate CART expression in terms of both transcript levels and immunoreactivity in caudal brain areas such as the locus coeruleus, NTS, PBN and the inferior olive (Douglass et al., 1995; Koylu et al., 1998), accorded with the increased Fos-IR identified in the NTS and PBN following i.c.v. CART into the LV (Vrang et al., 1999b). Comparable to i.c.v. injections into the LV or 3V, hindbrain delivery of CART peptide through the 4th ventricle (4V) led to reduction in food intake and body weight in both fed and food-deprived rodents, whilst the hypophagic effects showed no specificity to nutrients from either chow, sucrose or a nutritionally complete liquid diet (Aja et al., 2001b, 2002; Zheng et al., 2001, 2002; Skibicka et al., 2009) (Table 1). Importantly, the extent of feeding inhibition appeared more potent with CART administered into the 4V compared with LV injections (Zheng et al., 2001), raising the speculation that the hindbrain may house the key mediator for the hypophagic effects of i.c.v. CART (Aja et al., 2001b). Foundation for the idea involved the postulate that the anorectic effects triggered by forebrain i.c.v. CART indeed reflected the outcome of CART diffusion into hindbrain sites via the cerebrospinal fluid (CSF) (Aja et al., 2001b). Surmised from the CART-IR observed in cell bodies and central terminals of vagal afferent neurons projecting to the GI tract, a potential functional role of CART in meal termination and satiety may effectuate at the level of the brainstem (Broberger et al., 1999; Zheng et al., 2002). In rat, vagotomy caused considerable reduction in *CART* mRNA expression in several CART fibers in the vagus nerve and viscero-sensory nodose ganglion (Broberger et al., 1999). To verify such proposition, cerebral aqueduct occlusion was performed with an aqueductal plug to interrupt the forebrain-hindbrain CSF flow, and CART was injected into the 3V or 4V (Aja et al., 2001b) (Table 1). Interestingly, cerebral aqueduct blockage markedly attenuated the anorectic effects of 3V CART, whilst suppression of food intake remained unchanged when receiving 4V CART injection, signifying the independence of hindbrain CART in producing anorexia (Aja et al., 2001b). In contrast, hindbrain processing may be required or responsible for mediating a hypophagic response following forebrain or interbrain i.c.v. CART, further reinforcing the role of the brainstem in manifesting CART-driven anorectic effects, as concordant with the aforementioned higher potency in feeding inhibition with 4V as opposed to forebrain or interbrain i.c.v. CART (Zheng et al., 2001). Specifically, on the assumption that the repressed ingestive behaviors following LV or 3V CART may attribute to hindbrain CART action, the observations resulted from obtrusion of CSF flow could offer a possible explanation for the opposite feeding effects of orexigenic and anorexigenic natures induced by direct hypothalamic subnuclei (Wang et al., 2000; Abbott et al., 2001; Kong et al., 2003; Smith et al., 2008; Hou et al., 2010) vs. hindbrain ventricular (Aja et al., 2001b, 2002; Zheng et al., 2001, 2002; Skibicka et al., 2009) CART administration respectively. Such phenomena promote reevaluation of the authenticity and proposed mechanisms involved in the hypophagia exhibited after forebrain ventricular CART detailed in other studies (Kristensen et al., 1998; Lambert et al., 1998; Vrang et al., 1999b; Larsen et al., 2000; Abbott et al., 2001; Aja et al., 2001a,b).

## Functional implications of CART deletion on energy metabolism from genetic interventions

In order to gain further insight into the functional consequences of reduced or absent CART expression, several knockout models have been generated and characterized (Asnicar et al., 2001; Couceyro et al., 2005; Elefteriou et al., 2005; Osei-Hyiaman et al., 2005; Wierup et al., 2005; Moffett et al., 2006; Steiner et al., 2006) (Table 2). The phenotypic effects resulting from *CART* gene targeting approaches have in part shown inconsistency between studies, however, displayed a general trend in promoting positive energy balance. This in overall terms is also similar with results obtained from our novel *CART* knockout (KO) model, which was generated from a conditional version that was crossed with an oozyte-specific Cre line. Collective evidence regarding the role of CART in energy homeostasis from a few representative studies conducted by independent research groups (Table 2) as well as in-house results will be discussed.

**Table 2:**
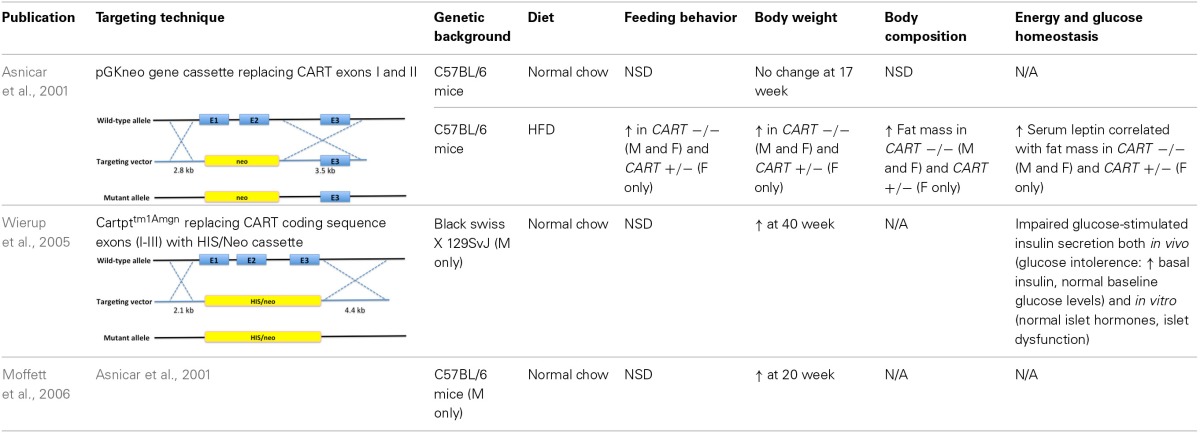
**Summary of the metabolic phenotypes of germline *CART* knockout mouse models in various studies when compared with wild-type controls**.

*Schematic representation of the gene targeting strategies adopted for generating CART knockout mouse models, depicting the wild-type allele of the mouse CART gene, the CART targeting vector, and the mutant CART allele. The three exons of the mouse CART gene are indicated by boxes labeled E1–E3. In the targeting constructs, the yellow boxes labeled “neo” or “HIS/neo” indicate the neomycin or histidinol/neomycin resistance selection cassettes respectively for targeted disruption of the CART gene. Homologous recombination between the targeting vectors and the complementary CART genomic loci (dotted lines) in mouse embryonic stem cells generates genetic ablations of exons E1 and E2, or all of exons E1–E3 of the CART coding region respectively. The targeting constructs were introduced into 129 SvJ mouse embryonic stem cells and subsequently injected into C57BL/6 blastocysts. M, male; F, female; HFD, high fat diet; NSD, no significant difference*.

In all models investigated, the expected increase in feeding due to depletion of the anorectic CART peptide was not observed under standard chow feeding conditions. However, when exposed to a high caloric diet, lack of CART led to altered feeding behavior and body weight (Asnicar et al., 2001; Couceyro et al., 2005; Wierup et al., 2005; Moffett et al., 2006). An increase in body weight has also been shown in *CART* KO mice fed on regular chow, although manifestation of such trait appears to require a longer time frame, where in one study, a statistically significant increase was absent at 17 weeks of age (Asnicar et al., 2001), whilst an independent group adopting the identical mouse model reported notably higher body weight for the knockout mice at 20 weeks (Moffett et al., 2006). In a different *CART* knockout mouse line, again altered weight was undetectable prior to 40 weeks of age (Couceyro et al., 2005; Wierup et al., 2005). Similarly, when fed a high fat diet, a significantly higher food consumption was observed in the *CART* KO compared to wild-type (WT) mice (Asnicar et al., 2001), in some but not all studies addressed. Importantly, despite a lack of consistent alteration in food intake, all studies reported an elevated body weight gain in *CART* knockout animals regardless of dietary options, with an even more prominent effect shown with a high caloric diet (Asnicar et al., 2001; Wierup et al., 2005; Moffett et al., 2006).

Interestingly, fasting-induced food intake experiments in our novel *CART* KO model suggest that lack of CART may be beneficial for body weight conservation during starvation. In our study, chow-fed *CART* KO animals showed a slightly lower food consumption compared with WT during a refeeding period following a 24-h fast, yet demonstrated a similar degree and pace of recovery in body weight, implying that less food might be required for returning to the pre-fast weight. On the other hand, the same *CART* KO mice on HFD experienced a less dramatic drop in body weight upon fasting, and accordingly showed a more effective weight regain to baseline during refeeding. Such improved reactions to food deprivation may be correlated to the observed difference in growth and possibly attributable to events in the interaction between CART and an improved stress response in *CART* KO mice, consistent with a role of the CART system in stress and anxiety-like responses (Koylu et al., 2000; Stanley et al., 2001, 2004; Kong et al., 2003; Larsen et al., 2003; Vrang et al., 2003; Dominguez et al., 2004a; Smith et al., 2004; Gozen et al., 2007; Hunter et al., 2007; Rogge et al., 2008; Nakhate et al., 2011). For example, i.c.v. CART has been reported to substantially influence the plasma levels of various stress hormones, such as adrenocorticotropic hormone and corticosterone (Stanley et al., 2001). Furthermore, anatomical implication has also been provided by the expression of CART transcript and peptide at various levels of the HPA axis as well as other stress-related areas in the CNS (Douglass et al., 1995; Couceyro et al., 1997; Koylu et al., 1997, 1998; Elias et al., 1998; Broberger, 1999; Larsen et al., 2003; Vrang, 2006; Rogge et al., 2008; Nakhate et al., 2011).

Amongst a multitude of potential factors contributing to the enhanced body weight in *CART* KO models, the gain in fat mass has been considered the most important (Asnicar et al., 2001). This is concordant with results from our novel *CART* KO model, where body composition analysis by dual-energy X-ray absorptiometry (DXA) revealed a pronounced increase in adiposity independent of diet. The gain in whole body fat mass was further confirmed by tissue dissection showing significantly elevated fat masses in all white adipose tissues, including the inguinal, epididymal, mesenteric and retroperitoneal regions. This also suggests an important function of CART in lipid metabolism, where CART has been linked to inhibition of lipogenesis and stimulation of lipid substrate mobilization and utilization (Rohner-Jeanrenaud et al., 2002; Wortley et al., 2004; Vasseur et al., 2007; Banke et al., 2013).

In addition to fat mass, lean mass constitutes another major determinant of energy homeostasis that directly influences energy expenditure. As opposed to the increase in fat mass, whole body lean mass of our CART-deficient mice was distinctly lower compared to WT controls regardless of dietary treatments. Similarly, the reduction was consistent across both periods of DXA measurements at 10 and 14 weeks of age. Intriguingly, research investigating the effects of chronic central CART infusion in genetically normal DIO rats reported a diminished body weight gain primarily due to a loss of lean mass (Larsen et al., 2000; Qing and Chen, 2007), while fat mass was unaffected (Qing and Chen, 2007).

Consistency exists between results from our *CART* KO model and previous studies focusing on the metabolic characterization of *CART* knockout mice, where no significant difference in total energy expenditure or physical activity could be detected when compared with WT controls regardless of diets (Asnicar et al., 2001; Moffett et al., 2006), even after the correction of the potentially confounding effects of lean mass on energy expenditure. However, overexpression studies have provided indications of both supporting and opposing roles of CART in regulating energy expenditure (Kong et al., 2003; Qing and Chen, 2007). An increase in energy expenditure was suggested in rats subject to chronic overexpression of CART through intra-arcuate targeted gene transfer, where the animals showed exaggerated weight loss and a downregulation of endogenous arcuate *CART* mRNA levels upon fasting and food restriction (Kong et al., 2003). Despite discordance between various animal studies, evidence exists for the involvement of CART in the regulation of energy expenditure and body weight in humans (Challis et al., 2000; del Giudice et al., 2001; Yamada et al., 2002; Dominguez et al., 2004a; Guerardel et al., 2005; Yanik et al., 2006; Rigoli et al., 2010). Specifically, a missense mutation in the pro-CART transcripts was discovered to co-segregate with a severe obese phenotype and was also associated with decreased resting energy expenditure in members of an Italian family over three generations (del Giudice et al., 2001; Dominguez et al., 2004a; Yanik et al., 2006).

In spite of a lack of direct indication endorsing a function of CART in modulating energy expenditure, comparing the respiratory exchange ratio (RER) between *CART* knockout animals and WT counterparts may shed light on any potential effects of CART ablation on energy metabolism through the fuel source preferences. Although no measurable difference was described in knockout animals in the respiratory quotient derived from RER as reported by previous studies (Asnicar et al., 2001; Moffett et al., 2006), a notably lower RER has been detected in our new *CART* knockout mice. Consistency in the lower RER particularly during the dark photoperiod was shown across both nutritional statuses, signifying that fat was preferentially metabolized over carbohydrates to supply energy for the body. A possible explanation for this could be the higher fat content in knockout animals, which may lead to the predominant fuel source based on the relative higher availability of fat than carbohydrates. On the other hand, a suppressed average respiratory quotient was demonstrated in both normal and DIO rats chronically overexpressing central CART compared to vehicle-treated controls (Rohner-Jeanrenaud et al., 2002; Qing and Chen, 2007). The reduction was exaggerated during the dark phase, under both regular feeding and fasting-refeeding conditions, indicating a stimulatory role of CART in promoting lipid oxidation and limiting fat storage, hence inhibiting excessive body fat accrual (Rohner-Jeanrenaud et al., 2002; Wortley et al., 2004; Qing and Chen, 2007).

Taken together, results from the literature as well as in-house studies of *CART* knockout models generally support the property of CART as a satiety factor and an anorexigenic signal in the brain, as evident in the elevation in body weight gain attributable mostly to the increased fat mass consistent across studies, although controversy exists for the corresponding food intake data. As for the aspect of energy intake, RER was demonstrated to be reduced both under the conditions of CART overexpression and ablation, suggesting fat was metabolized as the primary fuel for energy supply. A possible reason could be that although CART may intrinsically promote the utilization of fat as the predominant fuel source for reducing energy intake, the effectiveness of CART deletion on the disturbance of lipid metabolism hence accumulation of adiposity may have surmounted the simultaneous CART deficiency-induced enhancement of energy intake, resulting in a net reduction in RER based on the readily available fat depots.

## Roles of CART in human

As introduced earlier, evolutionary conservation has been demonstrated for CART in the neuroendocrine system across various mammalian species in the contexts of isoform structure, expression distribution pattern as well as functional implications, including a role of CART in the regulation of energy balance in human (Hager et al., 1998; Challis et al., 2000; del Giudice et al., 2001; Yamada et al., 2002; Dominguez et al., 2004a; Guerardel et al., 2005; Yanik et al., 2006; Rigoli et al., 2010). First, a genome-wide scan for human obesity-susceptibility loci in obese French Caucasian families (Hager et al., 1998) revealed a clear linkage to the chromosomal locus of 5q13.2 where the human *CART* gene is encoded (Table 3). Respectively, the expression of CART transcripts and peptides has been characterized in various hypothalamic areas involved in appetite control (Charnay et al., 1999; Elias et al., 2001; Menyhért et al., 2007), as well as in the subcutaneous and visceral white adipose tissues central to the moderation of lipid homeostasis (Vasseur et al., 2007; Banke et al., 2013). Intriguingly, the aforementioned anatomical-functional implications provided by the expression patterns of CART in the human infundibular nucleus, which demonstrated colocalization with the orexigenic NPY/AgRP and segregation from the anorexigenic POMC neurons, had rendered a primary anorectic role of CART appealable (Menyhért et al., 2007).

**Table 3:** **Examples of human studies demonstrating the association between genetic variations in the *CART* gene and the development of obesity**.

**Publication**	**Genetic association study**	**Ethnicity (sample size)**	**Body weight (sample size)**	**Genetic variation/susceptibility locus**	**Occurrence**	**Feeding behavior and body weight alterations**	**Biochemical alteration**	**Energy and glucose homeostasis**
Hager et al., 1998	Genome-wide scan for human obesity-susceptibility loci using model-free multipoint linkage analysis	French Caucasian (514)	Overweight (72), obese (107), morbidly obese (196), and non-obese controls (139)	Chromosomal locus 5q13.2 (*CART* gene)	Higher allele frequencies in overweight and obese sibpairs	N/A	Linkage with ↑ serum leptin levels	↑ Fasting glucose and insulin levels
Challis et al., 2000	Mutational analysis and population genetics	British Caucasian (902)	Morbidly obese (91) and non-obese (811)	1475A>G SNP (3′-UTR of exon 3)	NSD in allele frequency between obese and control subjects	Potential link to early-onset obesity; ↓ waist-to-hip ratio in male heterozygotes	Potential interference with fat distribution and contribution to dyslipidaemia	↓ Fasting plasma insulin and fasting triglycerides in male heterozygotes
del Giudice et al., 2001	Single-strand conformation polymorphism and automatic sequencing	Italian (230)	Obese (130) and non-obese controls (100)	Leu34Phe missense mutation in pro-CART (729G>C in exon 2)	A large family of obese subjects across three generations	Hyperphagia and severe early-onset obesity even when heterozygous for allele	Altered post-translational processing; intracellular missorting of proCART; bioactive CART deficiency in the serum; ↑ serum leptin levels	↓ Resting metabolic rates; linked to type II diabetes
Yamada et al., 2002	Single-strand conformation polymorphism and direct sequencing	Japanese (558)	Overweight and obese (528), non-obese controls (30)	6 polymorphic sites at 5′-flanking region, e.g., −156A>G [corresponds to −175A>G (Guerardel et al., 2005)], −929G>C	Higher allele frequencies in obese subjects than controls	↑ Genetic predisposition to obesity when in linkage disequliibrium	N/A	Potential association with type II diabetes
Guerardel et al., 2005	Sequence variability screen and haplotype analysis	French Caucasian (660)	Morbidly obese (292) and non-obese controls (368)	1475A>G SNP (3′-UTR of exon 3)	Higher allele frequencies in morbidly obese subjects than controls	N/A	N/A	N/A
Guerardel et al., 2005	Sequence variability screen and haplotype analysis	French Caucasian (989)	Morbidly obese (621) and non-obese controls (368)	5′ SNPs: −3608T>C, −3607C>T, −1702C>T, −175A>G; 3′UTR SNP: ΔA1457	Higher allele frequencies in morbidly obese subjects than controls; association enhanced with the SNP haplotype structure 3608T>C (or 175A>G) and −1702C>T, combined to ΔA1457	N/A	N/A	N/A
Guerardel et al., 2005	Sequence variability screen and haplotype analysis	French (2340) and Swiss (385) Caucasian	Moderately obese (619), morbidly obese (1006) and non-obese controls (1100)	−3608T>C SNP (promoter region)	Higher allele frequencies	↑ Genetic predisposition to obesity	Potential modulation of nuclear protein binding affinity	N/A
Vasseur et al., 2007	Sequence variability screen and haplotype analysis	French Caucasian (840)	General population sample	5′ SNPs: −3608T>C, −1702C>T, −175A>G (promoter region)	NSD in allele frequency between subjects with different BMI; strong linkage disequilibrium between the SNPs, haplotypic effect attributed to −3608T>C	N/A	↓ Plasma LDL-cholesterol level and LDL/HDL ratio; potential protection against atherogenesis	Potential association with lipid metabolism and atherogenicity
Rigoli et al., 2010	Family-based association methods	Italian (320)	Overweight (103), obese (30) and non-obese controls (187)	1475A>G SNP (3′-UTR of exon 3)	Higher allele frequencies in overweight (0.07) and obese (0.08) children compared to non-obese unrelated controls (children and/or adults) (0.02); preferential transmission of 1475G allele from heterozygous parents to overweight and obese offspring	Early-onset obesity	N/A	N/A

*With slight variation between different studies, body weight is categorized according to the body mass index (BMI): non-obese (<25 kg/m^2^), overweight (25–30 kg/m^2^), moderately obese (30–40 kg/m^2^), morbidly obese (>40 kg/m^2^). LDL, low-density lipoprotein; HDL, high-density lipoprotein; NSD, no significant difference; SNP, single nucleotide polymorphism; UTR, untranslated region; Δ, deletion*.

In human, alterations in *CART* have been associated with reduced metabolic rate, hyperphagia, obesity and elevated incidence of type II diabetes (Banke et al., 2013) (Table 3). For example, a Leu34Phe missense mutation in human proCART was discovered in obese members of an Italian family across three generations to affect post-translational processing, which coincided with CART peptide deficiency in the sera and reduced resting energy expenditure, ultimately leading to hyperphagia and severe early-onset obesity (del Giudice et al., 2001; Dominguez et al., 2004a; Yanik et al., 2006). In brief, the mutation was identified to neighbor a cluster of basic amino acids, hence presupposed to influence the specific processing of the proCART (1–89) in generating the biologically active CART I (42–89) and CART II (49–89) peptides (Kuhar and Yoho, 1999; Thim et al., 1999; del Giudice et al., 2001; Dominguez et al., 2004a). To simulate cellular effects of the mutation, subsequent investigations transfected human *CART* cDNA constructs representing either the wild-type or the mutant into corticotropic AtT-20 cells, a mouse pituitary-derived cell line often used for studying peptide processing and, more importantly, is known to express and process CART peptides (Dominguez et al., 2004a). Notably, in addition to reduced CART peptide levels in cells transfected with the mutated cDNA compared with controls, expression of the mutated proCART was also described to be mis-sorted, poorly processed and secreted, thus disarranging the cellular distribution of CART as a whole (Dominguez et al., 2004a). Other than discoveries that addressed the potentially crucial role of protein biosynthesis in the development of obesity, the majority of the studies directed at the association between CART and obesity focused on polymorphisms in *CART* within populations.

Polymorphism studies in the *CART* gene conducted worldwide have established substantial linkage between a few specific single nucleotide polymorphisms (SNPs) to obesity (del Giudice et al., 2001; Yamada et al., 2002; Guerardel et al., 2005; Rigoli et al., 2010) (Table 3). For instance, a family-based association study of 133 Italian trios has identified significantly higher allele frequencies of the 1475A>G SNP in the *CART* gene in overweight and obese children compared to non-obese unrelated controls consisting of both adults and children, while preferential transmission of the allele to overweight or obese children from heterozygous parents was predicted (Rigoli et al., 2010). In another Caucasian population, 292 French morbidly obese subjects were recruited for sequence variability screen in the *CART* gene, where a few SNPs residing in the promoter region, with SNP-3608T>C in particular, were suggested by haplotype analysis to prominently contribute to the genetic risk for obesity (Guerardel et al., 2005). The proposed association was further strengthened by the high prevalence of the specific allele in an expanded genotyping study, with additional populations of European Caucasian origin comprising 619 moderately obese French subjects and 385 morbidly obese Swiss subjects (Guerardel et al., 2005). Extended on the genetic studies, plausible functional effects of the SNP were also investigated by electrophoretic mobility shift assays in cellular system, where modulation of nuclear protein binding affinity was demonstrated to potentially correlate with the obesity phenotype (Guerardel et al., 2005). Besides Caucasians, a sequencing study in 528 Japanese subjects revealed a high level of polymorphisms in the 5′-flanking region of the *CART* gene housing the putative promoter region, wherein specific polymorphic sites or variants in linkage disequilibrium with each other were identified to associate with genetic predisposition to obesity (Yamada et al., 2002).

As mentioned, CART has recently been defined as a component of adipocytes involved in lipid substrate utilization in both human and rodents (Banke et al., 2013). Investigation in a large Caucasian population of approximately 1000 subjects in the United Kingdom identified two common polymorphisms in the 3′-untranslated region of *CART*, that were implicated to interfere with fat distribution and contribute to dyslipidaemia (Challis et al., 2000; Rogge et al., 2008) (Table 3). Despite a lack of correlation or consistent association with obesity through systematic mutational analysis (Lambert et al., 1998; Challis et al., 2000; Okumura et al., 2000; Rogge et al., 2008), a particular genetic variant 1475A>G among the polymorphisms was illustrated to significantly affect waist-to-hip ratio as well as the levels of plasma insulin and triglycerides (Challis et al., 2000), suggesting a putative pivotal role of CART in glucose- and lipid-homeostasis. Consolidation of the proposition has been illustrated in subsequent haplotypic study in a general population of 840 subjects from northern France (Vasseur et al., 2007), a continuum from the former French project on *CART* promoter SNPs (Guerardel et al., 2005), where three of the previously identified SNPs were described to affect plasma low-density lipoprotein-cholesterol level and consequently associated with cholesterol metabolism and atherogenicity (Guerardel et al., 2005; Vasseur et al., 2007). Specifically, the functional SNP-3808C>T was of particular interest, as plasma lipid profile traits protective against atherogenesis were displayed in cases bearing the allele, exemplifying the clinical potentials of *CART* in lipid metabolism and atherogenesis (Vasseur et al., 2007; Banke et al., 2013).

Taken together, human studies based principally on genetic polymorphisms have provided evidence promoting a role of CART in body weight regulation in humans. Altered *CART* expression has generally been associated with an elevated genetic predisposition to overweight and obesity, indirectly substantiating the anorexigenic nature of the peptide, although results from the literature show both anorexigenic and orexigenic properties of CART in animal studies. It is also noteworthy to address the plausible challenges imposed on the translatability of results obtained from animal models to the human system, considering the discernible difference in the anatomy of central CART-containing neurons between the two, as discussed above. Furthermore, although overall support has been gained for the hypothesis that inherited variations in *CART* could influence the development of obesity, such genetic linkage was absent for some other sequence variants detected within the gene, where the polymorphisms have been speculated as insufficient to disturb the peptide structure or create topological and conformational changes in the protein that would ultimately affect the functional activity of the peptide (Echwald et al., 1999; Walder et al., 2000; Rogge et al., 2008). Indeed, recent studies conducting an alanine scan for assessing the importance of the structure-activity relationship of CART demonstrated the dependence of anorexigenic potency on individual disulfide bridges in the peptide (Maixnerova et al., 2007; Maletinska et al., 2007; Blechova et al., 2013). To elucidate the contribution of specific disulfide bridges to maintaining the stability and biological function of CART, analogs with only one or two among the three disulfide bridges in the intact peptide were synthesized, with which binding activities as well as metabolic effects were measured in both cell and animal systems (Maixnerova et al., 2007). Intriguingly, results from binding experiments in PC12 rat pheochromocytoma cells (Maixnerova et al., 2007; Maletinska et al., 2007; Lin et al., 2011) indicated that the preservation of two particular disulfide bridges as well as the full-length peptide was imperative for biological activity, where high affinity of the analog to PC12 cells in both states of native phenotype and differentiated into neurons was measured (Blechova et al., 2013). In mice subjected to i.c.v. administration of the same analog, strong and long-lasting anorexigenic potency was exhibited during food consumption and behavioral tests, further purporting that one particular disulfide bridge could be omitted without a loss of bioactive function (Blechova et al., 2013).

In summary, the familial nature of obesity is well-established to be interrelated with a prominent genetic component. The CART system has been evident to constitute a dominant player in feeding control, body weight regulation and energy metabolism, hence a promising candidate for the development of anti-obesity therapeutics. Respectively, population genetics have revealed the potential contributions of polymorphisms in the *CART* gene to abnormalities in feeding and body weight control, where effects on interactions between the transcription factors and regulatory elements binding to the polymorphic sites may exert phenotypic influence. However, elucidating the mechanisms of CART action as well as investigating and replicating the fine genetic mapping in further populations will be essential for unraveling the authentic role of CART in energy homeostasis and understanding obesity.

## Conclusion

In conclusion, the widely adopted role of CART in the regulation of energy homeostasis has been summarized in this review from the perspectives of genetic and transcriptional associations, anatomical-functional correlation, pharmacological and genetic intervention studies in animal models, as well as implications from sequence variability in human. Nevertheless, the lack of a known CART receptor(s) and specific antagonists, continues to constitute the major challenge in understanding the underlying mechanisms by which CART exerts effects on feeding and neuroendocrine regulation.

The physiological importance of CART is endorsed by the evolutionarily conserved expression patterns in the brain as well as the periphery at regions associated with energy balance, inferring structural hence functional conservation in glucose sensing, lipogenesis regulation and ultimately the control of feeding behavior. The resultant integrated outcome on metabolic modulation also represents the neuronal crosstalk involving similar neuromodulatory peptides between central CART-interfered pathways residing at the hypothalamic and brainstem neurons as well as via the hypothalamic-pituitary-adrenal axis. In comparison with CART in the brain, functional roles of peripheral CART are less established, with indeterminate modes of action speculated to entail either a local independent response, or synergistic effects with central CART.

Overexpression of central CART through i.c.v. injections at either the forebrain (LV), interbrain (3V) or hindbrain (4V) areas has conformed to an anorexigenic role of CART, while the appetite-promoting effects of CART administered into specific neuronal targets may be attributed to a role of hypothalamic CART in stimulating the local release of orexigenic neuropeptides in a hypothalamic nucleus-specific manner. The discrepancy in anorexigenic and orexigenic circuitry highlights the major pitfall of non-specific widespread effects associated with i.c.v. ligand delivery, and was recognized through the efforts to identify potential sites for CART action following the analysis of Fos expression pattern representative of i.c.v. CART-induced neuronal activities. Candidate sites that may house the key mediator for the hypophagic effects of i.c.v. CART primarily reside in various feeding-related areas, including major hypothalamic nuclei, specific brainstem structures, and the Acb of the cerebral striatum. The appetite-inhibiting and -promoting effects produced by intra-accumbal CART and *CART* siRNA respectively reflect a concerted response from the CART-dopaminergic system interaction within the Acb, hence the observed feeding modulation and locomotive effects were likely perplexed by the inherent reward and motivation pathway. Several hindbrain areas are of particular interest as alternative brain regions for CART-induced anorexic effects, owing to the well-described role of the hindbrain in conveying post-prandial satiety effects to the hypothalamus, consonant with the indicated relation between CART and vagally-mediated gastrointestinal satiety.

In parallel with overexpression approaches, independent research groups have adopted genetic ablation to generate several germline *CART* knockout murine models, to determine effects of CART depletion on feeding behavior and metabolism. General consistency has been demonstrated between results from the literature and in-house data from our novel *CART* KO model, supporting CART as a satiety factor. Collectively, the phenotypic effects of CART deficiency from birth, although inconsistent across studies, display promotion toward positive energy balance. Extended findings stemming from our novel KO model also denote that CART deficiency may confer an advantage in body weight conservation during starvation, potentially associated with an improved stress response attributable to an altered anxiety-related cascade involving the CART system.

Both gene knockout and overexpression studies of CART have consolidated the stimulatory role of CART in promoting lipid oxidation and inhibiting fat accrual. As discussed above, despite the widely documented anorectic evidence of CART, possibility of an orexigenic role is indisputable. Under certain circumstances, such postulation may provide explanation for the inconsistent or lack of marked phenotypes in terms of energy balance observed in germline *CART* knockout animals, as well as conditional, developmental stage, or whole brain *CART* knockout models. Nevertheless, temporal or tissue-specific genetic ablation serves an invaluable means to minimize common pitfalls associated with a germline knockout strategy, such as any secondary effects or compensatory mechanisms during development.

In human, the expression of CART transcripts and peptides has been characterized in various hypothalamic areas involved in appetite control and lipid homeostasis. From the perspective of population genetics, genome-wide study of human obesity-susceptibility loci revealed linkage to a locus harboring the human *CART* gene, whereas mutation profiling has associated alterations in *CART* with reduced metabolic rate and resting energy expenditure, hyperphagia, obesity and elevated incidence of type II diabetes. Intriguingly, whilst a role of CART in regulating energy expenditure remains equivocal in overexpression studies and considered discordant in gene deletion approach in animal models, mutation screening in humans has consolidated the involvement of CART in the modulation of energy expenditure, body weight and dyslipidaemia.

To elucidate the underlying cellular mechanisms, functional effects of mutations and polymorphisms that were linked to the development of obesity were investigated. The sequence alterations of interests were described to potentially interfere with the post-translational processing of CART, leading to defective protein biosynthesis and deranged cellular distribution of the peptide, while modulation in the nuclear protein binding affinity was also reported to attenuate biological activity. Despite the plausible challenges imposed on the translatability of principal observations from animal models to the less well-documented human system, aberrations in CART have been demonstrated in human studies to promote positive energy balance, endorsing a primary anorectic role of CART.

In sum, the CART system remains a dominant player in the regulation of feeding, body weight and energy metabolism, hence a promising candidate for the development of anti-obesity therapeutics. However, elucidating the underlying mechanisms of CART action, developing relevant pharmacological tools and understanding the nature of the endogenous CART receptor(s), remains crucial in unraveling the functional role of CART in energy homeostasis and obesity.

### Conflict of interest statement

The authors declare that the research was conducted in the absence of any commercial or financial relationships that could be construed as a potential conflict of interest.
